# Machine Learning-Driven Biomarker Discovery for Skeletal Complications in Type 1 Gaucher Disease Patients

**DOI:** 10.3390/ijms25168586

**Published:** 2024-08-06

**Authors:** Jorge J. Cebolla, Pilar Giraldo, Jessica Gómez, Carmen Montoto, Javier Gervas-Arruga

**Affiliations:** 1 Takeda Farmacéutica España S.A., 28046 Madrid, Spain; carmen.montoto@takeda.com; 2 FEETEG, 50006 Zaragoza, Spain; giraldocastellano@gmail.com; 3 Hospital QuirónSalud Zaragoza, 50012 Zaragoza, Spain; 4 Anaxomics Biotech S.L., 08007 Barcelona, Spain; jessica.gomez@anaxomics.com; 5 Takeda Development Center Americas Inc., Cambridge, MA 02142, USA; javier.gervas@takeda.com

**Keywords:** Gaucher disease, GD1, skeletal complications, bone complications, biomarker, early diagnosis

## Abstract

Type 1 Gaucher disease (GD1) is a rare, autosomal recessive disorder caused by glucocerebrosidase deficiency. Skeletal manifestations represent one of the most debilitating and potentially irreversible complications of GD1. Although imaging studies are the gold standard, early diagnostic/prognostic tools, such as molecular biomarkers, are needed for the rapid management of skeletal complications. This study aimed to identify potential protein biomarkers capable of predicting the early diagnosis of bone skeletal complications in GD1 patients using artificial intelligence. An in silico study was performed using the novel Therapeutic Performance Mapping System methodology to construct mathematical models of GD1-associated complications at the protein level. Pathophysiological characterization was performed before modeling, and a data science strategy was applied to the predicted protein activity for each protein in the models to identify classifiers. Statistical criteria were used to prioritize the most promising candidates, and 18 candidates were identified. Among them, PDGFB, IL1R2, PTH and CCL3 (MIP-1α) were highlighted due to their ease of measurement in blood. This study proposes a validated novel tool to discover new protein biomarkers to support clinician decision-making in an area where medical needs have not yet been met. However, confirming the results using in vitro and/or in vivo studies is necessary.

## 1. Introduction

Gaucher disease (GD) is a rare autosomal recessive disorder that is considered a complex molecular storage disease characterized by the accumulation of sphingolipids, mainly glucosylceramide (GlcCer) [1]. It is caused by a deficiency in the activity of the enzyme glucocerebrosidase (GCase; EC 3.2.1.45), which leads to an accumulation of GlcCer, mainly in the monocyte-macrophage lineage, inducing phenotypic changes in Gaucher cells (GCs) [2]. This lipid buildup can lead to many symptoms and complications, ranging from bone pain and fatigue to anemia, neurological features, and organ dysfunction. GD is equally prevalent in males and females, with a worldwide prevalence of 0.70 to 1.75 per 100,000 individuals and 0.9 (95% CI 0.7–1.1) [3]. 

The disease is a consequence of pathogenic variants in the glucocerebrosidase gene (*GBA1*) (MIM*606403) and can be classified into three clinical subtypes based on the age of onset, clinical signs, rate of progression, or absence of neurologic disease [4]. Type 1 disease (GD1) (MIM#230800) has the most common onset in childhood or adulthood. The prevalence of GD1 is 0.26 to 0.63 per 100,000 individuals, which is considerably greater in the Ashkenazi Jewish population (estimated at 1 in 450 births) [3]. The most common clinical manifestations in GD1 patients are splenomegaly and hepatomegaly (present in up to 90% of patients). Other symptoms that may appear include anemia, thrombocytopenia, fatigue, and bone and joint pain [5,6]. GD1 disease can cause serious long-term complications if not properly diagnosed and treated. Particularly noteworthy is the significant impact of bone disease, which affects more than 80% of patients with GD1 studied, representing one of the complications with the most significant impact on quality of life [7,8]. 

The pathophysiology of GD1 disease results from the accumulation of GlcCer in macrophage lysosomes, especially in the spleen, liver, and bone marrow (BM), leading to chronic inflammation and cellular dysfunction [6,9]. GC infiltration into the BM and osteoclasts has been associated with increased osteoclastic activity and decreased osteoblastic activity [10]. GC infiltration at the BM level can contribute to the activation of T cells and the production of inflammatory cytokines, which can cause osteoclastic activation and bone resorption [11]. These processes can lead to decreased hematopoiesis, avascular necrosis (AVN), fragility fractures, and loss of bone density [12,13,14]. On the other hand, there is a scientific consensus for the classification of bone infarcts derived from these processes as avascular necrosis that can occur in the metaphyses or diaphyses of long bones and osteonecrosis in the epiphyses [15]. In addition, patients with GD1 are more susceptible to developing bone disease. These phenotypic variations of the disease are due to genetic factors and the proinflammatory effect of cytokines induced by GCs. All these components influence bone metabolism and bone marrow dysfunction in GD1 patients [16,17].

Early diagnosis and prompt intervention with specific therapies for GD are key in avoiding bone complications as much as possible [14,18,19]. The progressive skeletal compromise in patients with this disease has led to the proposal of an increasing number of follow-up recommendations during treatment to reduce associated morbidity and decreased quality of life [20,21,22]. Nevertheless, despite long-term therapy, several patients develop new bone crises, osteopenia, and other skeletal complications [23]. However, osteopenia can occur at any age. Despite treatment, some patients may continue to develop complications due to bone comorbidities. In addition, in a low percentage of GD1 patients, splenectomy is performed due to the enlargement of the spleen as a result of the accumulation of GCs; consequently, the bone disease becomes more aggressive [24,25]. Therefore, it is important to monitor the progression of the disease to predict morbidity [26].

The well-established monitoring biomarkers used for GD1 include chitotriosidase (ChT), glucosylsphingosine (Lyso-Gb1), the cytokine CCL18/PARC, angiotensin-converting enzyme (ACE), isoenzyme 5b of acid phosphatase (TRAP5b) and ferritin, among others. However, these methods have limitations in detecting bone involvement. Other biochemical markers related to bone, such as osteoclast-activating cytokines [16], MIP1-α, and MIP1-β [27], have been studied. Imaging studies, such as plain radiology, computed tomography (CT), dual-energy X-ray absorptiometry (DXA), or magnetic resonance imaging (MRI), may also be performed. These methods are critical tools for diagnosing and identifying skeletal involvement in GD patients. MRI is the *gold standard* for assessing bone marrow infiltration and identifying bone complications, such as AVN, bone infarcts, and bone crises [2]. However, no image or biochemical marker is currently available to detect prompt bone complications before irreversible complications occur at the skeletal level, constituting an unmet medical need [27,28]. 

Machine learning and artificial intelligence are bur GSE21899ning fields within biological and medical research, utilized to discern recurring patterns in complex datasets. These methodologies have found applications in the field of lysosomal diseases. For instance, they have been employed to identify patients at risk of developing Fabry disease by assessing the presence of phenotypic patterns in a patient’s clinical data [29]. Furthermore, they have been used to unearth potential biomarkers of early tissue damage in metachromatic leukodystrophy [30]. This emergent technology has also found utility in the study of more prevalent diseases such as hypertrophic cardiomyopathy. This is evidenced by the work conducted by You H. et al., where potential genes were identified as biomarkers [31]. Focus on GD1, this technology allowed the assessing of changes in bone microstructure [32] and the identification of characteristics of patients susceptible to developing bone complications during treatment [19]. Moreover, systems biology-based approaches can help to detect promising biomarkers for precisely diagnosing diseases [33,34] and untangle complex pathological states, especially in rare disease areas [35,36], such as GD, where information is scarce [37,38].

Therefore, the main objective of this project was to identify protein biomarkers capable of accurately predicting the early diagnosis of bone complications in GD1 patients using novel tools, such as machine learning methods.

## 2. Results

### 2.1. GD1 Disease Characterization, Interactome and Mathematical Models

The models were trained on a compendium of biological and clinical data describing human physiology (Supplementary Table S1).

**Figure 1:**
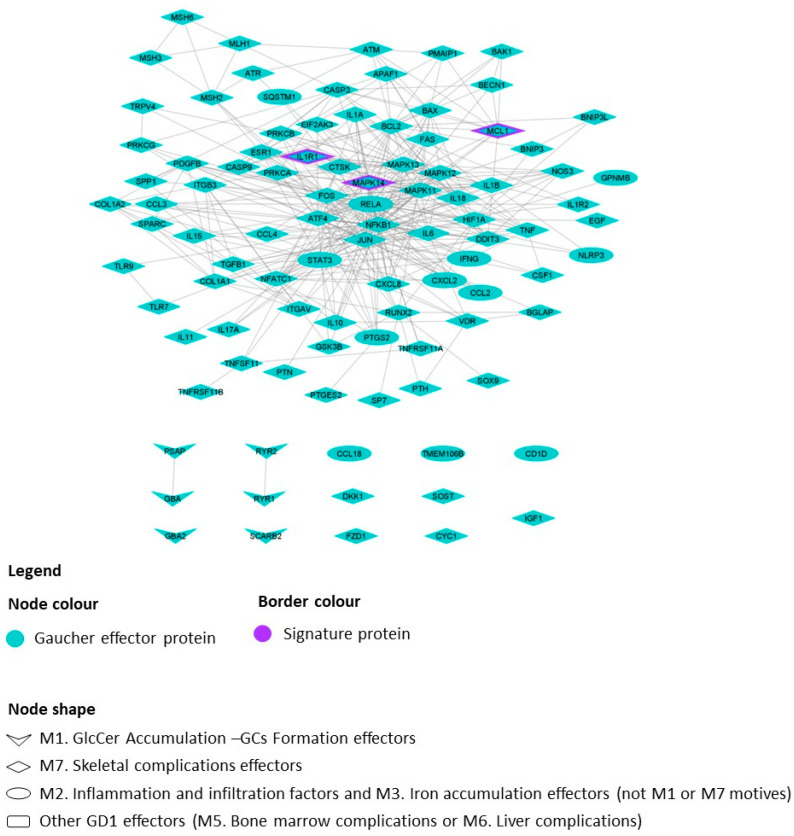
Graphical representation of the GD1 protein network, including skeletal complication effectors and their interactions with the selected protein effectors, as well as the most promising biomarker candidates and their interactions. The image presents directional link information between proteins, including all available relationships (links, gray color) between proteins (nodes, blue color) from a regularly updated in-house database drawn from public sources. The shape of the nodes indicates the GD1 motif to which the depicted proteins belong, and the purple border indicates the proteins belonging to the GD1 protein expression signature. GD1: type 1 Gaucher disease. GlcCer: Glucosylceramide. GCs: Gaucher Cells. M#: the number of identified pathophysiological processes or motifs involved in GD1.

To focus the models on GD1, we used its molecular characterization derived from a nonsystematized literature review, as noted in the methods section (Supplementary Table S2). We focused our analysis on a set of 7 pathophysiological processes, or motifs (M), and 150 single protein effectors (Table 1, Supplementary Table S2) found to be involved in GD1 disease pathophysiology. The effectors were integrated into the global human protein–protein interaction network (Figure 1) for a human physiology-based evaluation of GD1.

We queried for publicly available GD1 gene expression datasets in gene expression data repositories. One study complied with the filters: expression data of cultured skin fibroblasts from five samples from untreated GD1 patients and four samples from healthy individuals in the GSE21899 series [39] available from GEO [40]. Exclusively considering genes mapping to proteins, we defined a GD1 protein expression signature (Supplementary Table S3).

The network of the proteins directly connected to GD1 contained 3809 proteins, which were found to be significantly enriched within the fibroblast expression-based GD1 protein expression signature (*p* value < 10^−4^). We evaluated how the skeletal complication-related proteins connected to the proteins within the initial pathological changes associated with GD1, including “GlcCer Accumulation–GC Formation”, “inflammation and infiltration factors” and “iron accumulation in GCs” motifs (Figure 1). We found that although there was a direct connection between “inflammation and infiltration factors” and “iron accumulation in GCs”, no direct interactions were found between the proteins and the “GlcCer Accumulation–GC Formation” motif.

Once the proteins involved in GD1 were identified, we aimed to identify the proteins most prone to triggering GD1 initiation. Although the “GlcCer Accumulation–GCs Formation” motif is the initial trigger of the disease [2], its lack of direct connectivity with the “inflammation and infiltration factors”, “iron accumulation” and “skeletal complications” motifs prevented us from using it as a direct trigger of the rest of the disease. Accordingly, given that GC formation leads to infiltration and subsequent manifestation [2], we evaluated which of the proteins affected by the “inflammation and infiltration factors” pathway were more prone to triggering the rest of this pathway and the “iron accumulation” pathway; both of these pathological changes subsequently lead to disease manifestation. To do so, we used pretrained therapeutic performance mapping system (TPMS) models [41] to obtain the number of proteins associated with the GD1 motifs “inflammation and infiltration factors” and “iron accumulation”, with the sign assigned at distances of 1, 2 and 3 for each “inflammation and infiltration factors” protein effector indicating that protein proximity was positively correlated. A mean value was obtained for the universe of solutions, and a normalized value was obtained considering the maximum signal that could be achieved (maximum value: 100%). We selected the effector group that allowed 100% of the remaining effectors to reach “inflammation and infiltration factors” and “iron accumulation” as a stimulus. We found that nuclear factor interferon gamma (IFNG), interleukin-6 (IL6) and macrophage colony-stimulating factor 1 (CSF1) were able to induce the protein expression of 100% of these genes in terms of the predicted protein activity signal.

To simulate GD1 with different complications, we created 8 TPMS models using the following components: (1) stimulus: the GD1 triggering identified stimulus; (2) restrictions: in addition to the training set (Supplementary Table S1), the GD1 protein expression signature, as previously described [41], was used to better model the pathophysiology of GD1; and (3) response: a combination of GD1-defined motifs (Table 2).

A total of eight models were created, each containing 250 mathematical model solutions, simulating the pathophysiology of patients with GD1 and its possible associated complications, including modeling GD1 without complications and modeling GD1 with individuals or combinations of skeletal, bone marrow and liver complications. The mean between these solutions provides the most likely paths from the stimulus to the response. In addition to the interactions that link stimulus and response, sample-based method models allow the exploration of how signals flow between proteins in the network and the predicted protein activity achieved for each protein (ranging from [−1 to 1]) within the models. This predicted protein activity can be used to analyze and compare the models.

The aim of the current study was to identify markers of skeletal involvement (defined as proteins involved in osteoblast/osteoclast uncoupling and hypoxia-induced osteonecrosis). To do so, the following comparisons were defined for identification:

- *Biomarker candidates for skeletal complications*: GD1 with skeletal complications model vs. the other models.
- *Biomarker candidates for bone marrow complications*: GD1 with bone marrow complication models vs. the other models.
- *Biomarker candidates for liver complications*: GD1 with liver complication models vs. the other models.

To prioritize specific biomarkers of skeletal complications, the candidates that also complied with the first three filters when considering *biomarker candidates for bone marrow complications* (GD1 with bone marrow complications models vs. the rest of the models) or *biomarker candidates for liver complications* (GD1 with liver complications models vs. the rest of the models) were discarded.

Although the TPMS models are protein based, the interactome in which they are built includes gene and RNA regulation data; thus, for standardization purposes, we will use gene names to refer to all genes/proteins mentioned in this manuscript when referring to the model results.

The models created allowed the exploration of the classification potential of 423 GD1-related proteins, 89,253 pairwise combinations between them and 12,525,171 combinations of 3 proteins to accomplish the goal of this project.

### 2.2. Evaluation of Previously Described Biomarkers

Previously proposed skeletal complication (such as osteonecrosis, bone damage, or metabolic changes in bone tissue) biomarkers in GD1 according to the literature were primarily chemokines: C-C motif chemokine 3 (*CCL3* gene, MIP-1α), cathepsin K (*CTSK* gene), C-C motif chemokine 4 (*CCL4* gene, MIP-1β), osteocalcin (*BGLAP* gene), interleukin-8 (*CXCL8* gene), C-C motif chemokine 18 (*CCL18* gene) and C-C motif chemokine 5 (*CCL5* gene). According to our models and the data science strategy applied, four out of the seven candidates showed statistically significant cross-validated balanced accuracy (cross-validated *p* value < 0.01) (Table 3). CCL4/MIP-1β was the previously proposed biomarker with higher cross-validated balanced accuracy (58.54%); thus, we set this value as the threshold for new potential candidate discovery. The validation process applied was leave-one-out (LOO) cross-validation. This validation consists of estimating the threshold value for all available samples, except the sample that is excluded from the analysis. The process is repeated, keeping a different sample out of the analysis each time, and the final threshold is determined as the average of all thresholds determined.

### 2.3. Identification of Potential Biomarkers of GD1-Related Skeletal Complications

Applying this cross-validated accuracy filter as well as precision and sensitivity (≥65%) filters, we identified 18 biomarker candidates (formed by 1, 2 or 3 proteins) that showed potential as *biomarker candidates for skeletal complications* and not as *biomarker candidates for bone marrow complications* or *biomarker candidates for liver complications* (Table 4). Sixteen of these biomarker candidates contained at least 1 protein measurable in plasma or urine, namely, CCL3/MIP-1α, interleukin-8, interleukin 1 receptor type 2 (*IL1R2* gene), platelet-derived growth factor B subunit (*PDGFB* gene) and parathyroid hormone (*PTH* gene) in plasma and G protein subunit alpha I3 (*GNAI3* gene) in urine.

Among these candidates, we identified combinations of previously proposed biomarkers with additional proteins, which increase their classification potential within our models. We identified two combinations improving CCL3/MIP-1α (in combination with the platelet-derived growth factor B subunit and induced myeloid leukemia cell differentiation protein Mcl-1 (*MCL1* gene) and in combination with the platelet-derived growth factor B subunit and parathyroid hormone) and two combinations improving interleukin-8 (in combination with PDGFB and MCL-1 in combination with GNAI3 and MCL-1). The CCL3/MIP-1α, PDGFB and PTH combination consisted of proteins that are easily measurable in plasma.

Considering proteins not previously proposed as potential *skeletal complication biomarkers* (Table 4), we identified a classifier formed exclusively by one protein, IL1R2, which is reported to be measurable in plasma. We identified 10 combinations of two proteins and 3 combinations of three proteins fulfilling all the filters. Among them, only the combination of IL1R2 with PDGFB is easily measurable in blood. Moreover, the addition of PDGFB increases the IL1R2 classification capability based on cross-validated accuracy, precision and sensitivity.

When considering the 18 biomarker classifiers, three GD1 effectors appeared repeatedly, either alone or as part of a combination with other proteins, in several of the candidates: IL1R2, MCL-1 and PDGFB.

**Figure 2:**
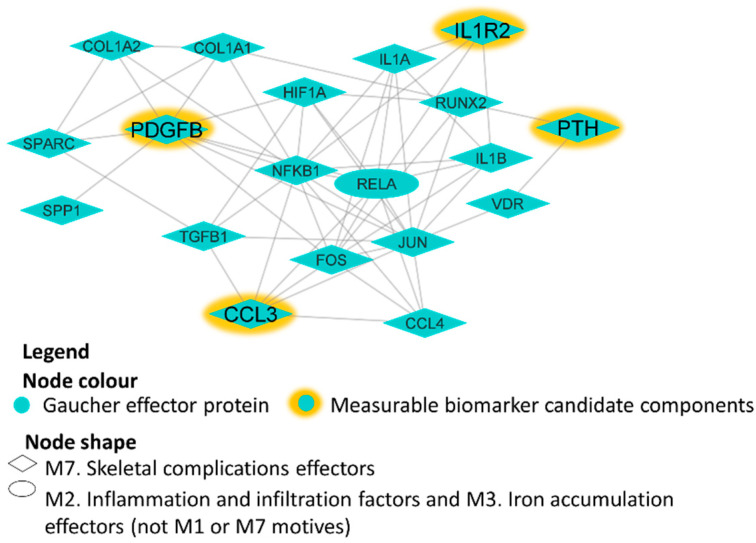
GD1 protein effectors around measurable biomarker candidate components. The image presents directional link information between proteins, including all available relationships (links, gray color) between proteins (nodes, blue color) and candidate biomarkers (nodes, yellow color) from a regularly updated in-house database drawn from public sources. The shape of the nodes indicates the GD1 motif to which the depicted proteins belong. M#: the number of identified pathophysiological processes or motifs involved in type 1 Gaucher disease; GD1: Type 1 Gaucher disease.

Of the 18 biomarker candidates, 4 were composed of elements all suitable for laboratory plasma measurements (Table 4): IL1R2, PDGFB, CCL3/MIP-1α and PTH. These four proteins, in addition to being GD1 protein effectors, are highly connected to the molecular characterization of GD1 (Figure 2).

## 3. Discussion

Models predicated on systems biology and machine learning represent innovative and potent tools for the study of rare diseases. These diseases often suffer from a paucity of data. Current recommendations, as suggested by several authors, underscore the necessity for in vitro and/or in vivo validation of all predictive models. This validation is crucial for their subsequent utilization for clinical or biomedical purposes [46]. In addition, rare disease populations are very heterogeneous compared to those with other diseases [35,36]. In the case of this study, our methodology is based on a well-established technology that has provided validated results in vitro and in vivo [47,48,49,50]. Emerging omics technologies for quantifying thousands of molecules, coupled with the sophisticated development of artificial intelligence algorithms, can accelerate biomarker discovery [51]. Many studies have shown that machine learning, versus classical methods, can better discriminate between healthy and diseased groups and identify essential biomarkers for use in clinical decision-making in various settings [52]. In addition, we observed that the theoretical interactome on which our model is based reflects enrichment in the observed expression differences between GD1 fibroblasts and healthy fibroblasts in publicly available expression datasets. In the present study, systems biology-based models have allowed us to propose the repurposing and combination of proteins as potential early diagnostic biomarkers related to skeletal complications in GD1 patients.

Currently, skeletal complications in GD1 patients are diagnosed using imaging approaches that quantify skeletal involvement once established [2,53]. The identification of biomarkers as predictive tools for skeletal complications in GD1 patients is an unmet medical need because imaging techniques, although good follow-up tools, do not allow the prediction of bone comorbidities, and the available bone biomarkers are insufficient [13]. The wide spectrum of clinical manifestations and their unpredictable progression make it necessary to use more precise biomarkers that facilitate early diagnosis and avoid irreversible complications [54]. Most patients with GD1 have lesions at the time of diagnosis, and this debilitating skeletal involvement affects their quality of life [55]. It would be desirable to identify biomarkers for early diagnosis in young patients to anticipate irreversible complications or perform more sensitive follow-up than imaging techniques.

The relationships between chemokines and cytokines and osteonecrosis in GD, as well as between chemokines and proinflammatory cytokines in GD1, have already been studied [11,16,56]. Several proinflammatory cytokines, including interleukin-1 beta (IL-1 beta), interleukin-6 (IL-6), tumor necrosis factor-alpha (TNF alpha) and interleukin-10 (IL-10), have been used to evaluate the systemic and local manifestations of GD [57]. In our study, we propose a list of 18 biomarkers, including some of the previously proposed markers (CCL3/MIP-1α, CXCL8), as well as the involvement of the proinflammatory state (IL1R2) [57,58]. Most of these proteins are defined as effectors, although they have not been described as markers of skeletal complications. Some of the proteins that we did not include in the characterization at the time should also be highlighted: isoform B of 1-phosphatidylinositol 4,5-bisphosphate phosphodiesterase beta-1 (*PLCB1* gene), sterol regulatory element-binding protein 1 (*SREBF1* gene), G protein subunit alpha I3 (*GNAI3* gene), and tyrosine-protein phosphatase nonreceptor type 11 (*PTPN11* gene). PLCB1 is typically used for the evaluation of bone regulation [59]. SREBF1 is associated with pathogenic changes in Parkinson’s disease and plays a role in lysosomal cholesterol accumulation [60], and changes in this biomarker have also been detected in the brains of GD1 model mice after treatment [61]. GNAI3 functions as a downstream transducer of G protein-coupled receptors (GPCRs) in numerous signaling cascades. Guanine and nucleotide binding protein 3 promotes odonto/osteogenic differentiation of apical papilla stem cells through the JNK and ERK signaling pathways, although one of the manifestations of GD1 patients includes delayed eruption of definite teeth [62]. Finally, PTPN11 acts downstream of various receptor and cytoplasmic protein tyrosine kinases to participate in signal transduction from the cell surface to the nucleus, and this process plays an essential role in osteoblast differentiation [63]. However, we did not find an association between any of these proteins and GD1 patients in the scientific literature.

To facilitate further studies and potential clinical translation, we highlighted those proteins within the biomarker candidates that have been shown to be measurable in plasma. There are four biomarker candidates that are composed of proteins that are all measurable: IL1R2, PDGFB, CCL3/MIP-1α and PTH. IL1R2 is found in neutrophils, B cells, monocytes, and macrophages. IL1R2 is a natural inhibitor of IL1, plays essential roles in inflammation and immune regulation [64,65], and is specifically involved in activating hematopoietic precursors in the BM [66]. The accumulation of glycolipids in GD1 contributes to the activation of different inflammatory mediators [6]. The regulation of IL1R2 expression in different cell types has been described as a mechanism to counteract exacerbated inflammation in response to exogenous stimuli. In the context of bone pathophysiology under inflammatory conditions, the exacerbated osteoclastic activity that results in bone loss is related to the IL1 response and IL1R2 expression [65].

PDGFB is responsible for the development of fibroblastic and myofibroblastic tumors [67]. PDGFB regulates stem cell-based bone regeneration [68]. Specifically, the PDGFB chain increases trabecular bone formation and trabecular connectivity, and decreases cortical porosity [69]. At the vascular level, it stimulates the thickening of the intima layer of blood vessels [70]. These proteins also play important roles in maintaining homeostasis and normal cell function. When this regulation is compromised, as occurs in GD1 disease [71], changes in cell metabolism, intracellular signaling, and other biological processes can occur that can alter these proteins.

CCL3/MIP-1α serves as a critical mediator of the immune response by attracting leukocytes to sites of inflammation. In patients with GD1, elevated CCL3/MIP-1α levels are correlated with the severity of bone involvement, suggesting a central role for this chemokine in the underlying pathological process [16,72,73]. Alterations in the inflammatory secretome of bone marrow mesenchymal stromal cells could contribute substantially to the skeletal pathology observed in patients with GD1 [74].

PTH is essential for bone metabolism to regulate calcium homeostasis and remodeling, mediating critical actions through its type-1 receptor (PTHR1) in bone development and mineral regulation [75,76]. In this context, osteocytes modulate the activity of sclerostin, a key inhibitor of bone formation, in response to PTH using posttranslational degradation mechanisms through lysosomal pathways, which are essential for bone formation induced by anabolic stimuli. Lysosomal dysfunction, which is characteristic of patients with GD1 disease, compromises this process, establishing a direct connection between bone alterations in the disease and PTH [77]. Additionally, teriparatide, a synthetic form of PTH (1–34), is an effective therapeutic strategy for improving bone mineral density and architecture in patients with GD1 disease who do not respond to bisphosphonate treatments [78].

This, together with the fact that all these genes are GD1 effectors and are connected to other players in the disease, make these results good candidates for further validation. Although our strategy is theoretical, the approach aims at minimizing biases by using available information from GD1 disease to train our artificial network and has adequate biological contextualization. Additionally, we used previously proposed biomarkers to set a threshold for the identification of good candidates. 

Within the limitations of our study, this is due to the gene/protein nature of the TPMS modeling approach, as other relevant biological molecules cannot be directly evaluated. In this sense, other approaches based on other biological molecules (lipidomics [79] or fibroblasts, for example) could complete our findings.

Additionally, there is a lack of information associated with this disease, and this limitation is especially relevant for rare diseases such as GD1. Although we compiled all available information and applied TPMS technology [41], which considers not only disease but also the entire landscape of human physiology, to reduce this limitation, there are other factors that our models might not have considered. In this study, we focused on proteins of skeletal involvement (involved in osteoblast/osteoclast uncoupling and hypoxia-induced osteonecrosis) and separated them from BM complications. BM complications are associated with depressed hematopoiesis characterized by an infiltrative phenomenon [13], which could explain why medullary pathology is reflected in the hematopoiesis process [2]. Although a vascular component is involved in the pathophysiology of BM and we cannot rule out local vascular phenomena, we excluded it from BM complications because the vascular factors involved are still unknown and their effect on BM organization and function remains unclear, as the infiltrative phenomenon is the most established process for BM pathology in GD [2]. We also consider GC infiltration a global process because we found no evidence of specific search mechanisms for different tissues; if there were diverse mechanisms, our models could have proposed non-skeleton-specific markers. To control for this bias, we discarded those candidates that also had the potential to rank model solutions for liver complications and complications due to BM infiltration.

Overall, we believe that machine learning-driven modeling can help deepen our understanding of the skeletal and bone marrow complications of GD1, as shown by our biomarker candidate results, which are consistent with current GD1 knowledge and could be readily evaluated in preclinical or clinical settings.

Consequently, our strategy has proposed panels of markers with the capacity for classification, repositioned previously proposed markers and combinations of proteins that could complement current strategies to promptly identify skeletal involvement in patients with GD1. Further research in this field must align with an in vitro and/or in vivo validation [46] in a cohort of patients who have a thorough clinical characterization of the pathology. This will allow for the validation of those markers that present a diagnostic and prognostic purpose related to the skeletal complications of GD1.

## 4. Materials and Methods

**Figure 3:**
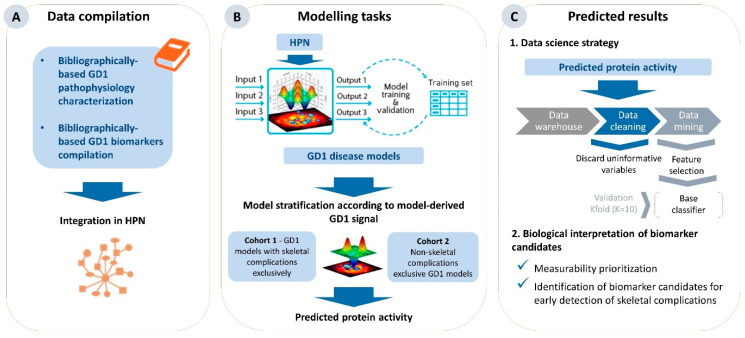
Workflow for the identification of potential biomarker candidates for GD1-related skeletal complications. This process involves three steps: (**A**) data compilation for molecular characterization of GD1 generation to develop the HPN; (**B**) modeling to generate GD1 mathematical models using a systems biology-based TPMS approach; and (**C**) data analyses employing model outputs, which were applied to the protein activity inferred from the mathematical models to classify and identify potential biomarker candidates. GD1: Type 1 Gaucher disease; HPN: human protein network; TPMS: Therapeutic Performance Mapping System.

An in silico study was developed using the TPMS patented by Anaxomics [41], which is based on systems biology, machine learning, and pattern recognition techniques that integrate all available biological, pharmacological, and medical knowledge to create mathematical models that simulate human pathology and physiology. The steps were as follows: data compilation (Figure 3A), modeling task (Figure 3B) and results prediction (Figure 3C).

### 4.1. Data Collection and Gaucher Disease Interactome Characterization

The first step in modeling GD1 was molecular characterization (Figure 3A). We reviewed the nonsystematized literature in the Medline/PubMed database. The search strategy is shown in Supplementary Table S4.

We evaluated the results at the title, abstract, and, if containing molecular information (i.e., proteins related to the pathophysiology of GD1), full-text level to identify the main pathophysiological processes or motifs involved in GD1. The search was expanded by checking relevant articles in the reference lists of the resulting articles. Subsequently, we identified proteins that are functionally associated with the development of GD1 through their activity and functions (or lack thereof), and these proteins were defined as protein effectors. After identifying potential candidate proteins, in those cases where the evidence found was not sufficiently consistent to be attributed to an effector (i.e., assigning a functional role for the protein in disease development), specific literature searches were performed using the Medline/PubMed database either to confirm or discard the candidate. This procedure has been previously applied in studies yielding experimentally validated conclusions [47,48,49,50]. 

Gene expression data were searched in the following databases: Gene Expression Omnibus (GEO) [40], ArrayExpress [80] and Omics Discovery Index (DI) [81]. The following search and filters were used: *“Gaucher” [ALL FIELDS] AND “Homo sapiens” [Organism]*; only transcriptomics experiments were considered. The selected data were processed with GEO2R [82,83]. The genes considered significantly differentially expressed between diseased and control fibroblasts were those with an adjusted *p* value ≤ 0.05 and an absolute value of log2FC ≥ 1. Exclusively considering genes mapped to proteins, we defined a GD1 protein expression signature (Supplementary Table S2).

Hypergeometric enrichment analysis [84] was applied to evaluate the presence of the GD1 protein expression signature within the bibliography- and database-based GD1 interactomes, using UniProtKB [42], THPA [43,44] and the Clinical Urine Proteomics Database [45] as the protein universe.

TPMS technology [41] was applied to build GD1 models considering the human map of known protein relationships described in the following databases: KEGG [85], REACTOME [86], INTACT [87], BIOGRID [88], HPRD [89] and TRRUST [90]. Using the compiled data, we created a network of human proteins (Figure 3A). Network representations were created using Cytoscape 3.8 [91].

### 4.2. Generation of Mathematical Models

This section shows the modeling process using mathematical models (Figure 3B). TPMS mathematical models are created using a whole human protein–protein interaction network that incorporates the available relationships (edges or links) between proteins (nodes) from a regularly updated in-house database drawn from public sources, as previously described [41,92]. To transform this network or map into mathematical models capable of both reproducing existing knowledge and predicting new data, it was trained on a compendium of biological and clinical data describing human physiology. This training set is defined as the input–output relationships between drug definitions and indications at the protein level. Therefore, the protein map derives a mathematical model similar to the multilayer perceptron (MLP) of an artificial neural network over the human protein network. To do so, each link in the network is assigned a weight, and the signal is propagated across the system starting from the input. Then, the sample-based methods allow the exploration of how the signal flows between the proteins in the network and the predicted protein activity reached for each protein (ranging from [−1 to 1]) within the models. This predicted protein activity can be used to analyze and compare the models.

Although the TPMS models are protein based, the interactome in which they are built includes gene and RNA regulation data; thus, for standardization purposes, we will use gene names to refer to all genes/proteins of the model results mentioned in the tables and figures.

### 4.3. Results Prediction

#### 4.3.1. Statistical Analysis: Classifier Identification

This section describes the data science strategy used to predict the model’s results (Figure 3C). Potential biomarkers based on single variables, pairs, and 3-variable combinations were identified using a data science strategy, comparing predicted protein activity derived from the models.

The previously described data science strategy [41] consisted of 3 steps: data cleaning, data mining and cross-validation. Data cleaning consisted of removing uninformative variables. Data mining included two steps. First, feature selection was performed for the evaluation of classifiers composed of 2 or 3 variables (1-variable classifiers were explored by brute force) by applying the following feature selection methods: CHOW-LIU [93], MRMR [94], RELIEFF [95], RFE-SVM [96], SFFS [97] and Wilcoxon with correlation [98]. Then, a base classifier was calculated either by identifying the optimal linear or quadratic threshold for classifiers composed of 1 variable or GLM binomial [99], naive Bayes [100] or MLPs for classifiers composed of 2 or 3 variables. Finally, a 10 K-fold cross-validation was applied. The cross-validated balanced accuracy was used as a classifier optimization measure together with the cross-validated *p* value.

To select the most promising protein candidates, the following filters were applied. First, we considered the capability of the *previously proposed biomarkers* as *biomarker candidates for skeletal complications* in terms of their cross-validated balanced accuracy (Equation (1)) in separating GD1 from the skeletal complications model vs. the other models; thus, only candidates improving the best performing *previously proposed biomarker* were retained. The BACC was calculated considering both sensitivity (how well the positives are predicted, i.e., cohort 1) and specificity (how well the negatives are predicted, i.e., cohort 2) as follows:

(1) $$BACC=\frac{\left(specificity+sensitivity\right)}{2}$$

To select those candidates that maximize the identification of solutions in the models of skeletal complications compared to the rest, we set thresholds to ensure a minimum of sensitivity but also precision (success rate when a sample is predicted as positive) and select candidates with a minimum of quality with respect to these parameters. Thus, relevant literature highlighting the importance of selecting balanced sensitivity and specificity thresholds was consulted as a second filter, especially in studies characterized by high clinical heterogeneity. This methodology was adopted to maximize diagnostic accuracy and clinical relevance for diverse populations, such as those with GD1 disease, establishing a sensitivity threshold ≥ 65% to ensure minimum diagnostic sensitivity and adequate precision [41].

The objective was to obtain a selection of candidates for the following evaluations. Third, candidate biomarkers that included an unmodulated protein in at least 75% of the model solutions from either of the two cohorts tested were excluded. Considering that the Gaucher population is very heterogeneous, this filter aims to identify proteins that are modulated homogeneously. Thus, it is relevant to measure the protein in any patient despite this heterogeneity.

All the simulations and computational processes described in this project were executed in the Anaxomics computing cloud, which integrates over 800 computational threads on machines with 64 gigabytes of RAM. The software, databases and tools used are from Anaxomics Biotech.

#### 4.3.2. Measurability Information

Additionally, to provide further information on the classifiers to obtain the most promising biomarker candidates from a clinical translation point of view, we used the information stored in dedicated databases. To obtain information on biomarkers detectable in plasma and urine, we explored The Human Protein Atlas (THPA) [43,44] (*Plasma protein* in Table 1) and the Clinical Urine Proteomics Database [45] (*Urine protein* in Table 1). 

## 5. Conclusions

An in silico model suggested repositioning known biomarkers and biomarker combinations for the early diagnosis of bone complications in GD1 patients using plasma measurements of IL1R2, PDGFB, CCL3/MIP-1α and PTH, although in vitro and/or in vivo validation is needed. Machine learning-based modeling may further our understanding of GD1 bone complications.

## Figures and Tables

**Table 1:** Summary of the motifs identified as involved in type 1 Gaucher disease.

M ID	M Name	Number of Proteins
M1	GlcCer accumulation–GC formation	6
M2	Inflammation and infiltration factors	24
M3	Iron accumulation in GCs	12
M4	Splenic complications	0 *
M5	Bone marrow complications	21
M6	Liver complications	43
M7	Skeletal complications (Osteoblast/osteoclast uncoupling)	43
	Skeletal complications (Hypoxia-induced osteonecrosis)	42

Splenic complications: increased sequestration of platelets. Bone marrow complications: depressed hematopoiesis. Liver complications: liver fibrosis/liver disease/liver disease and fibrosis. Skeletal complications: vascular involvement in bone disease/bone turnover dysfunction. * The precise molecular mechanisms by which platelets are sequestered in the spleen remain unknown. Thus, this motif cannot be defined at the molecular (protein) level. GlcCer: Glucosylceramide. GCs: Gaucher Cells. M#: the number of identified pathophysiological processes or motifs involved in type 1 Gaucher Disease.

**Table 2:** Type 1 GD models and combinations of motifs used for response.

TPMS Model Name	Response (GD1 Motif Combination)
GD1 without complications	M2. Inflammation and infiltration factors M3. Iron accumulation in GCs
GD1 with skeletal complications	M2. Inflammation and infiltration factors M3. Iron accumulation in GCs M7. Skeletal complications
GD1 with liver complications	M2. Inflammation and infiltration factors M3. Iron accumulation in GCs M6. Liver complications
GD1 with BM complications	M2. Inflammation and infiltration factors M3. Iron accumulation in GCs M5. BM complications motif
GD1 with BM and liver complications	M2. Inflammation and infiltration factors M3. Iron accumulation in GCs M5. BM complications M6. Liver complications
GD1 with liver and skeletal complications	M2. Inflammation and infiltration factors M3. Iron accumulation in GCs M6. Liver complications M7. Skeletal complications
GD1 with BM and skeletal complications	M2. Inflammation and infiltration factors M3. Iron accumulation in GCs M6. BM complications M7. Skeletal complications
GD1 with BM, liver and skeletal complications	M2. Inflammation and infiltration factors M3. Iron accumulation in GCs M5. BM complications M6. Liver complications M7. Skeletal complications

Splenic complications: increased sequestration of platelets. Bone marrow complications: depressed hematopoiesis. Liver complications: liver fibrosis/liver disease/liver disease and fibrosis. Skeletal complications: involvement in bone disease/bone turnover dysfunction. BM: Bone Marrow. GD1: Type 1 Gaucher disease. GCs: Gaucher Cells. M#: the number of identified pathophysiological processes or motifs involved in GD1. TPMS: Therapeutic Performance Mapping System.

**Table 3:** Classification performance of the biomarkers of skeletal complications that were previously proposed by third authors.

Protein Name	Gene Name [40]	UniProt ID [42]	crosVal Accuracy (%)	crosVal *p*-Value
MIP-1β	*CCL4*	P13236	58.54	9.32 × 10^−32^
Cat K	*CTSK*	P43235	58.4	6.72 × 10^−41^
MIP-1α	*CCL3*	P10147	53	0.000218
OC	*BGLAP*	P02818	51.31	2.83 × 10^−10^

The classification was performed in terms of cross-validated balanced accuracy to separate the GD1 skeletal complication models from the other GD1 models. Only candidates showing crosVal-Accuracy > 50% are shown. For the creation of the in silico model, the gene nomenclature was used, because the model includes protein and genetic regulation relationships. Cat K: cathepsin; GD1: Type 1 Gaucher disease; OC: osteocalcin; MIP-1α: C-C motif chemokine 3; MIP-1β: C-C motif chemokine 4.

**Table 4:** The new potential classifier candidates were ranked from lowest to highest based on their cross-validated percentage-balanced accuracy.

Gene Name [40]	UniProt ID [42]	crosVal Accuracy (%)	crosVal Precision (%)	crosValSensitivity (%)	crosVal *p*-Value	Plasma Protein [43,44]	Urine Protein [45]
*MCL1*	Q07820	66.69	65.32	71.14	3.62 × 10^−89^	No	No
*PLCB1*	Q9NQ66					No	No
*JAK2*	O60674	66.80	65.46	71.14	2.37 × 10^−90^	No	No
*MCL1*	Q07820					No	No
*IL1R2*	P27930	67.54	68.25	65.60	7.26 × 10^−98^	Yes	No
*PDGFB*	P01127	68.31	65.04	79.20	1.97 × 10^−112^	Yes	No
*PTH*	P01270					Yes	No
***CCL3***	**P10147**					Yes	No
*PDGFB*	P01127	68.54	66.30	75.43	1.51 × 10^−111^	Yes	No
*PLCB1*	Q9NQ66					No	No
*GNAI3*	P08754	70.11	72.25	65.31	4.77 × 10^−130^	No	Yes
***CXCL8***	**P10145**					Yes	No
*MCL1*	Q07820					No	No
*PDGFB*	P01127	70.26	67.18	79.20	1.62 × 10^−135^	Yes	No
*SREBF1*	P36956					No	No
*IL1R2*	P27930	70.77	72.15	67.66	5.66 × 10^−138^	Yes	No
*MCL1*	Q07820					No	No
*PDGFB*	P01127	70.97	71.92	68.80	2.29 × 10^−140^	Yes	No
*IL1R2*	P27930					Yes	No
*IL1R2*	P27930	71.54	69.41	77.03	4.62 × 10^−150^	Yes	No
*SREBF1*	P36956					No	No
*IL1R2*	P27930	73.14	70.19	80.46	6.74 × 10^−176^	Yes	No
*PLCB1*	Q9NQ66					No	No
*JAK2*	O60674	73.83	70.44	82.11	2.45 × 10^−188^	No	No
*IL1R2*	P27930					Yes	No
*PDGFB*	P01127	74.00	76.12	69.94	3.55 × 10^−186^	Yes	No
*MCL1*	Q07820					No	No
*PDGFB*	P01127	74.20	78.03	67.37	2.58 × 10^−192^	Yes	No
*MCL1*	Q07820					No	No
***CCL3***	**P10147**					Yes	No
*PDGFB*	P01127	75.20	77.43	71.14	4.96 × 10^−206^	Yes	No
*PTPN11*	Q06124					No	No
*MLC1*	Q07820					No	No
*PDGFB*	P01127	76.26	79.82	70.29	8.24 × 10^−227^	Yes	No
*SREBF1*	P36956					No	No
*MCL1*	Q07820					No	No
*PDGFB*	P01127	76.54	77.86	74.17	2.31 × 10^−228^	Yes	No
***CXCL8***	**P10145**					Yes	No
*MCL1*	Q07820					No	No
*PDGFB*	P01127	77.26	79.96	72.74	2.15 × 10^−243^	Yes	No
*GNAI3*	P08754					No	Yes
*MCL1*	Q07820					No	No

Previously proposed skeletal complication biomarkers are highlighted in bold, and whether the protein components of the candidates are measurable in plasma or urine according to dedicated databases is indicated. For the creation of the in silico model, gene nomenclature has been used because the model includes protein and genetic regulation relationships.

## Data Availability

Data contained within the article.

## Supplementary Materials

The following supporting information can be downloaded at https://www.mdpi.com/article/10.3390/ijms25168586/s1. Reference [101] is cited in the supplementary materials.
